# Cognitive Impairment After Stroke: A Multifaceted Challenge in Search of Personalized Solutions

**DOI:** 10.3390/brainsci15121301

**Published:** 2025-12-01

**Authors:** Pasquale Moretta, Simona Spaccavento

**Affiliations:** 1 Istituti Clinici Scientifici Maugeri IRCCS, Department of Neurorehabilitation, Institute of Telese Terme (BN), 82037 Telese Terme, Italy; 2 Istituti Clinici Scientifici Maugeri IRCCS, Department of Neurorehabilitation, Institute of Bari, 70124 Bari, Italy

Stroke remains one of the leading causes of adult disability worldwide, with cognitive impairment representing one of its most devastating and persistent consequences [1,2]. Beyond motor and sensory deficits, post-stroke cognitive dysfunction profoundly affects quality of life, social integration, and long-term independence [3]. The studies collected in this Special Issue, “Cognitive Impairment and Related Complications of Stroke: Prevention, Diagnosis, and Treatment,” underscore the multifaceted nature of cognitive and clinical outcomes after stroke, exploring the neurobiological underpinnings, clinical correlates, and potential therapeutic strategies for cognitive recovery. Collectively, they offer a nuanced view of the mechanisms, assessment tools, and interventions that can shape more effective prevention and rehabilitation strategies for stroke survivors.

The paper “A Comparative Study on Cognitive Assessment in Cerebellar and Supratentorial Stroke” provides a compelling starting point for this discussion. Traditionally, the cerebellum has been viewed primarily as a structure involved in motor coordination, yet mounting evidence reveals its crucial role in cognitive and affective processes. This study demonstrates that cerebellar infarction leads to cognitive deficits resembling those observed in frontal lobe damage—particularly in attention and executive functions—though to a milder degree. Importantly, the study identifies the Addenbrooke’s Cognitive Examination III (ACE-III) and the Cerebellar Cognitive Affective Syndrome scale (CCAS-s) as sensitive tools for detecting subtle cognitive dysfunction in cerebellar stroke. These findings not only reinforce the concept of a “cerebellar cognitive affective syndrome” but also expand our understanding of cerebro-cerebellar circuitry in higher cognition. The recognition that cerebellar lesions can yield cognitive profiles akin to frontal dysfunction challenges conventional diagnostic frameworks and emphasizes the importance of tailored neuropsychological assessment across all stroke topographies.

Moving from diagnosis to intervention, “Neuropsychological Performance after Extended N-Pep-12 Dietary Supplementation in Supratentorial Ischemic Stroke” offers encouraging evidence for the potential of nutritional strategies to enhance post-stroke cognitive recovery. In this randomized controlled trial, extended supplementation with the neuropeptide-based compound N-Pep-12 led to significant improvements in global cognition, particularly in Montreal Cognitive Assessment (MoCA) and processing speed scores, over a one-year period. The favorable safety profile further underscores the feasibility of this intervention as an adjunct to conventional rehabilitation. These findings point toward an emerging paradigm where neurorestorative nutrition may complement pharmacologic and behavioral approaches in facilitating neural plasticity and recovery. While replication and larger-scale trials are needed, this study provides an important step toward personalized, multimodal rehabilitation strategies that address the biological substrates of cognitive resilience after stroke.

The complexity of stroke-related cognitive impairment becomes even more evident when examined through the lens of systemic and genetic disorders, as illustrated by “Prevalence and Clinical Correlates of Cerebrovascular Alterations in Fabry Disease: A Cross-Sectional Study.” Fabry disease, a lysosomal storage disorder, represents a model for understanding the interplay between vascular pathology and cognitive decline. This study reveals that mild-to-moderate cerebral small vessel disease (CSVD) is common in Fabry disease and closely linked to renal impairment, highlighting the systemic nature of the vascular pathology. Cognitive impairment affected nearly 70% of the cohort, emphasizing that cerebrovascular damage in Fabry disease is not merely structural but functionally consequential. The study’s exploration of serum neurofilament light chain (NfL) as a biomarker of cerebral small vessel disease is particularly noteworthy. Although preliminary, the findings suggest that NfL might serve as a minimally invasive marker of neuroaxonal damage, potentially bridging the gap between clinical presentation and the underlying pathology. The study thus exemplifies how research on rare diseases can yield insights into the broader mechanisms of cerebrovascular cognitive impairment, offering potential biomarkers and targets applicable across aetiologies.

A different yet equally intricate dimension of post-stroke cognition is explored in “Novel Insights into Viewer-Centered Versus Stimulus-Centered Hemispatial Neglect: A Cross-Sectional Behavioral and Imaging Study of Acute Stroke.” Hemispatial neglect, a disabling condition affecting spatial awareness, remains a paradigmatic model of stroke-induced cognitive dysfunction. This large-scale imaging and behavioral study dissects the neural substrates of two distinct neglect subtypes—viewer-centered and stimulus-centered—and reveals that each arises from specific lesion patterns and hypoperfusion territories across both hemispheres. Crucially, the study challenges the long-held notion that neglect is predominantly a consequence of right hemisphere damage, showing that stimulus-centered neglect is also common after left hemisphere strokes. By integrating lesion load with perfusion data, the authors demonstrate that both structural and functional disruption contribute to neglect, emphasizing the value of early imaging markers in predicting and managing this syndrome. These results hold direct implications for acute stroke care, where understanding the precise neural mechanisms of neglect may guide targeted reperfusion therapies and improve functional outcomes.

The next two contributions turn the focus toward language and communication—domains that epitomize the intersection of cognition, function, and identity after stroke. In “Neural Bases of Language Recovery After Stroke Can Only Be Fully Understood Through Longitudinal Studies of Individuals,” a conceptual and methodological shift is advocated. The author argues that traditional group-averaged neuroimaging studies obscure the true variability in recovery trajectories, as each individual’s brain reorganizes through unique neural pathways. Through a synthesis of longitudinal imaging studies, the paper demonstrates that mechanisms of language recovery vary dramatically even among patients with similar lesion profiles, reflecting the interplay of structural damage, compensatory reorganization, therapeutic interventions, and premorbid brain health. The central message is clear: meaningful insights into post-stroke language recovery demand longitudinal, individualized approaches. Averaging across subjects, while statistically appealing, risks erasing the very heterogeneity that defines human neuroplasticity. This perspective invites a rethinking of both research methodology and clinical rehabilitation—encouraging the personalized mapping of recovery rather than one-size-fits-all assumptions.

Complementing this theoretical insight, “Clinical and Linguistic Correlates of Functional Communication Abilities after Stroke: A Longitudinal Study” grounds the discussion in practical outcomes. Studying patients with post-stroke aphasia during neurorehabilitation, the authors identify comprehension abilities and basic functional autonomy as key predictors of recovery in everyday communication. Improvements were observed across all language domains and in overall functional independence, yet the regression analyses revealed that neither demographic factors nor general clinical variables significantly predicted communication gains. Instead, receptive language and activities of daily living emerged as the strongest determinants of functional recovery. This finding highlights the deeply interconnected nature of linguistic, cognitive, and motor processes in post-stroke rehabilitation. Moreover, it underscores the necessity of ecologically valid assessment tools—those that capture communication in real-life contexts rather than abstract linguistic tasks. Such evidence has important implications for designing rehabilitation programs that address both the cognitive and functional dimensions of recovery.

Taken together, the six studies featured in this Special Issue chart an integrative landscape of stroke-related cognitive impairment—from cerebellar to cortical lesions, from systemic genetic disorders to focal ischemia, and from mechanistic imaging studies to interventional trials. Several overarching themes emerge. Firstly, cognitive impairment after stroke is not confined to any single brain region or mechanism; rather, it reflects a network-level dysfunction encompassing cortical, subcortical, and cerebellar circuits. Secondly, effective diagnosis requires sensitive and specific tools such as ACE-III, CCAS-s, and MoCA, as well as imaging- and biomarker-based approaches that capture subtle and dynamic changes in brain function. Thirdly, recovery is inherently individualized—shaped by neural plasticity, systemic health, environmental context, and targeted intervention. Finally, emerging evidence suggests that recovery can be facilitated not only through rehabilitation and pharmacotherapy but also through nutritional and behavioral strategies that support neuronal resilience.

In conclusion, the contributions to this Special Issue collectively advance our understanding of the cognitive and communicative consequences of stroke. They reveal a future where prevention, diagnosis, and treatment are informed by a holistic appreciation of brain–behavior relationships, enriched by biomarkers, imaging, and personalized interventions. Cognitive impairment after stroke is no longer viewed as an inevitable aftermath but as a dynamic and modifiable condition—one that, through scientific insight and clinical innovation, can be addressed with growing precision and hope.
